# Imperforate Hymen

**Published:** 1874-08

**Authors:** T. M. Shaw

**Affiliations:** Coosa, Floyd county, Georgia


Imperforate Hymen.—I was called to see a girl, aged fifteen, the afternoon of the 22nd of December, 1873. At the time of my arrival she was suffering intensely with pain in the lower portion of abdomen and small of the back, at times greater than at other times, resembling labor pains. I ascertained from her mother that she had suffered periodically in the same way for two years past, but that at this time she was suffering more than usual. On placing my hand over the left iliac region, I felt a tumor the size of my fist, smooth, compressible, movable, and very painful to the touch. Suspecting some menstrual trouble, I obtained a digital examination of the vagina, and found the trouble to be imperforate hymen. There was not the slightest opening. On perforating the hymen, from one to two pints of menstrual blood escaped, the tumor in the iliac region disappearing instantly, and the girl relieved without any outward result.

The above is at your disposal. Respectfully,

T. M. SHAW, M. D.

COOSA, Floyd county, Georgia.

